# Urinary Protein-to-Creatinine Ratio: An Indicator of Adverse Clinical Outcomes in Preeclampsia With Proteinuria

**DOI:** 10.7759/cureus.23341

**Published:** 2022-03-20

**Authors:** Arzoo Chadha, Surekha Tayade

**Affiliations:** 1 Department of Obstetrics and Gynaecology, Jawaharlal Nehru Medical College, Datta Meghe Institute of Medical Sciences (Deemed to be University), Wardha, IND

**Keywords:** hypertension, eclampsia, proteinuria, urinary protein creatinine ratio, preeclampsia

## Abstract

Background and objective

Preeclampsia is a major contributor to morbidity and mortality among pregnant women and leads to poor fetomaternal outcomes. Predicting fetal and maternal health outcomes will enable early interventions so as to reduce further damage. Various biochemical tests like beta-human chorionic gonadotropin (β-HCG), inhibin A, activin A, pregnancy-associated plasma protein-A (PAPP-A), fetal DNA, and color Doppler have been studied for their ability to predict fetal and maternal health outcomes; however, most of these tests are complex and costly. Among the many variables that indicate the severity of outcomes in hypertensive disorders of pregnancy, the urinary protein-to-creatinine ratio (UPCR) is an important index. The aim of the study was to find out the association between UPCR and fetomaternal outcomes in preeclampsia.

Material and methods

A prospective observational study was conducted among 141 women with preeclampsia presenting with proteinuria, who were divided into two groups: 11% with UPCR <0.3 and 89% with UPCR ≥0.3. These patients were followed up till delivery to look for maternal and fetal outcomes.

Results

The sensitivity of UPCR for predicting adverse maternal outcomes was 79.37% (95% CI: 71.25-86.06), specificity was 46.67% (95% CI: 21.27-73.41), positive predictive value (PPV) was 92.59% (95% CI: 88.53-95.29), negative predictive value (NPV) was 21.21% (95% CI: 12.43-33.81), and the accuracy was 75.79% (95% CI: 67.97-82.69); for adverse fetal outcomes, the sensitivity was 76.98% (95% CI: 68.65-84.01), specificity was 13.33% (95% CI: 1.66-40.46), PPV was 88.18% (95% CI: 85.69-90.29), NPV was 6.45% (95% CI: 1.79-20.67), and the accuracy was 70.21% (95% CI: 61.94-77.62).

Conclusion

Based on our findings, UPCR is a simple laboratory tool that can help predict abnormal fetomaternal outcomes in preeclampsia with good sensitivity and PPV and can be used as an adjunct to assist in clinical decisions.

## Introduction

Pregnancy is a physiological state associated with substantial and profound alterations in metabolic and biochemical processes, most of which are reversible following delivery. The phrase maternal-fetal medicine refers to a treatment approach that aims to successfully treat both sides of the maternal-fetal unit, i.e., health concerns of the mother and fetus before, during, and shortly after pregnancy. Unfortunately, many of the disease conditions seen in obstetrics necessitate treating one side of the maternal-fetal unit at the expense of the other. Hypertensive diseases of pregnancy are the best example of this duality. Hypertensive disorders have long been recognized as an important complication of pregnancy [1]. They continue to be a leading cause of fetal and maternal morbidity and mortality worldwide [2,3]. Hypertensive disorders complicate 5-10% of all pregnancies, especially in developing countries like India where 14-16% of maternal deaths are reportedly due to hypertensive disorders [4].

Preeclampsia is a pregnancy-specific hypertension syndrome. The American College of Obstetricians and Gynecologists defines the diagnostic criteria for preeclampsia as the measurement of hypertensive thresholds (i.e., systolic and diastolic blood pressures ≥140 and ≥90 mmHg, respectively, occurring twice, four hours apart, after 20 weeks) with either proteinuria (i.e., ≥300 mg per 24 hours) or, in the absence of proteinuria, new onset of any of the following systemic findings: (a) thrombocytopenia (platelet count <100,000 µL); (b) renal insufficiency (i.e., creatinine >1.1 mg/dL or two-fold increase in creatinine in the absence of underlying renal disease); (c) abnormal liver function (i.e., hepatic transaminase levels twice the upper limit of normal); (d) pulmonary edema; or (e) cerebral or visual symptoms [5].

Of note, 3-8% of all pregnancies worldwide are affected by preeclampsia [6,7]. The exact pathophysiology of preeclampsia is not completely known. Endothelial dysfunction, which is regarded to be a major factor for multiorgan failure, affects critical organs such as the kidney, liver, and brain, resulting in a variety of adverse maternal and fetal consequences [8]. Therefore, it is critical to look for predictive markers for at-risk pregnancies that can indicate poor maternal or fetal outcomes. If we are able to predict the disease course, it can enable early detection of maternal and fetal disorders, as well as the timely dispensing of appropriate management protocols, ensuring that the pregnancy ends with a healthy mother and child. A non-invasive test with a good predictive value entails categorizing pregnant women depending on their risk of disease progression so that women can be advised of appropriate antenatal follow-up depending on their risk status.

Proteinuria levels are important for obstetricians to consider when making clinical decisions regarding delivery in preeclamptic women [9]. It is important to note that urinary protein excretion increases during pregnancy and total protein excretion of more than 300 mg in a 24-hour urine collection sample is deemed abnormal [10]. In preeclampsia, proteinuria is recognized as an independent risk factor and predictor of end-organ damage. The identification of an increase in protein excretion, in particular, is recognized to have both diagnostic and prognostic value, and proteinuria measurement can be critical in determining disease progression [11]. The urinary protein-to-creatinine ratio (UPCR) normalizes protein excretion to the glomerular filtration rate and therefore remains unaffected by hydration status [12]. While the gold standard method for quantifying proteinuria is 24-hour urine protein [13], this important procedure has a certain time limit for detection. Furthermore, it is inconvenient, prone to collection errors, and is associated with low patient compliance. The detection of proteinuria with a random UPCR test, which has been extensively studied and is suggested to have better accuracy, feasibility, and provides faster results than 24-hour urinary protein excretion, is a simple replacement tool that can be used effectively in place of 24-hour urine collection [14,15].

## Materials and methods

**Figure 1:**
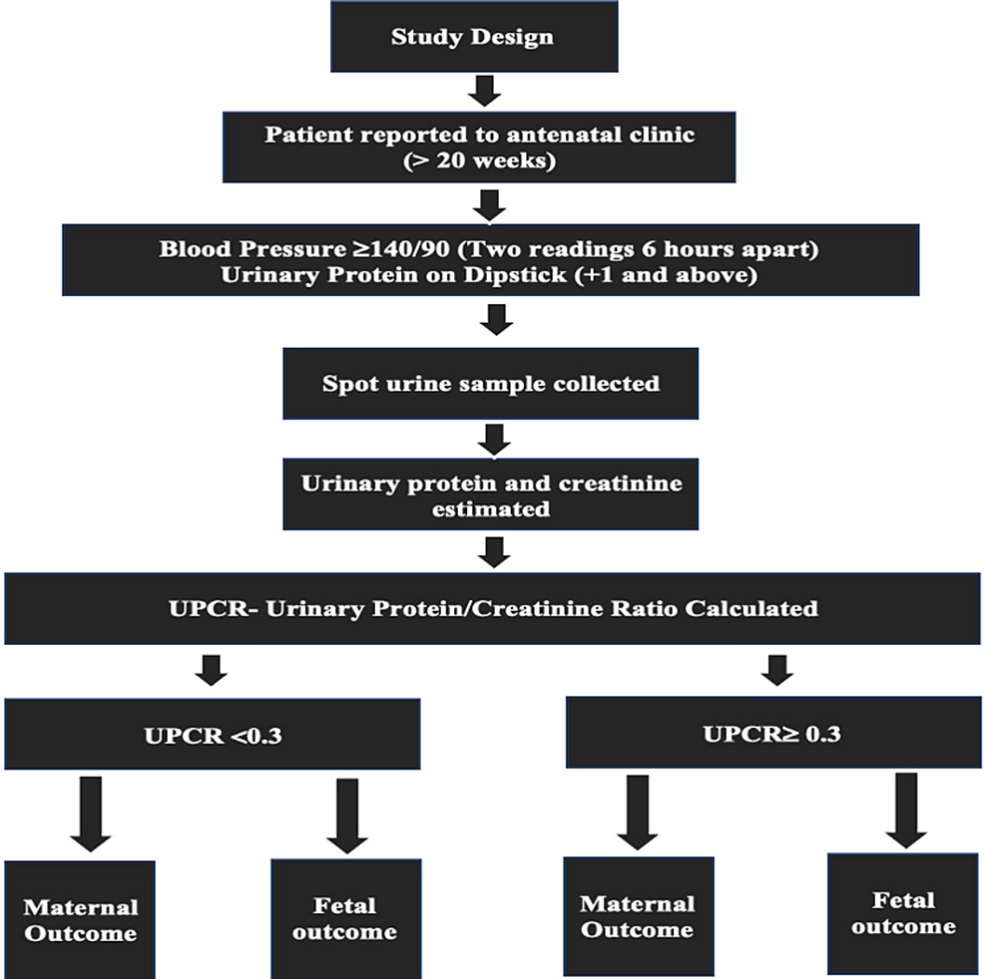
Schematic representation of the conduct of the study UPCR: urinary protein-to-creatinine ratio

Our main aim was to study UPCR and its association with fetomaternal outcome in preeclampsia associated with proteinuria. This prospective, hospital-based, observational study was carried out from September 2019 to September 2021 (two years) at the Datta Meghe University of Medical Sciences, Wardha, India. The target population included 152 pregnant women seeking care in the outpatient and inpatient units of the Department of Obstetrics and Gynaecology at the study site, who were deemed eligible based on inclusion criteria and amenable to follow-up till delivery (Figure 1).

Selection criteria for subjects for the study

The inclusion criteria were as follows: pregnant women seeking antenatal care at the study site and having a blood pressure equal to or greater than 140/90 mmHg on two occasions (six hours apart) with proteinuria (dipstick +1 and above) after 20 weeks of gestation and willing to give informed written consent to be a part of the study.

Ethical approval

The ethical approval for the study was obtained from the Ethical Review Committee of the Datta Meghe University of Medical Sciences, Wardha (Deemed to be University). The approval letter was numbered DMIMS(DU)/IEC/2021/563.

Operational definitions

Maternal Parameters

New episode of severe hypertension (≥160/110 mmHg) [16].

Renal insufficiency (serum creatinine >1.2 mg/dL) or oliguria (<400 mg/dL) [17].

Increased liver enzyme [aspartate aminotransferase (AST) >40 u/l] [18].

Presence of thrombocytopenia (platelet count <150 x 10^9^/l) [19].

HELLP (Hemolysis, Elevated Liver Enzymes, and Low Platelets) syndrome [20].

Abruptio placentae: hemorrhage where the bleeding occurs due to premature separation of normally situated placenta [21].

Fetal Parameters

Prematurity: a baby born before 37 completed weeks of gestation calculated from the first day of the last menstrual period [22].

Fetal growth restriction (FGR): birthweight below the 10th percentile of the average for the gestational age [23].

Low birth weight (LBW) of newborns: babies that weigh less than 2500 gm at birth [24].

Intrauterine death (IUD): babies with no signs of life in utero after 24 weeks of gestation [25].

Statistical analysis

The data were recorded in the proforma, and it was transferred to a Microsoft Excel spreadsheet. Qualitative data were expressed in the form of percentages while quantitative data were expressed as mean ± SD and range values. The relationship between qualitative data was tested using the Chi-squared or Fisher’s exact test, and the independent sample t-test was used for quantitative data. A p-value <0.05 was considered statistically significant. Sensitivity, specificity, positive predictive value (PPV), negative predictive value (NPV), and diagnostic accuracy of UPCR were calculated. The data were subjected to statistical analysis using the SPSS Statistics software version 24.0 (IBM, Armonk, NY).

## Results

**Figure 2:**
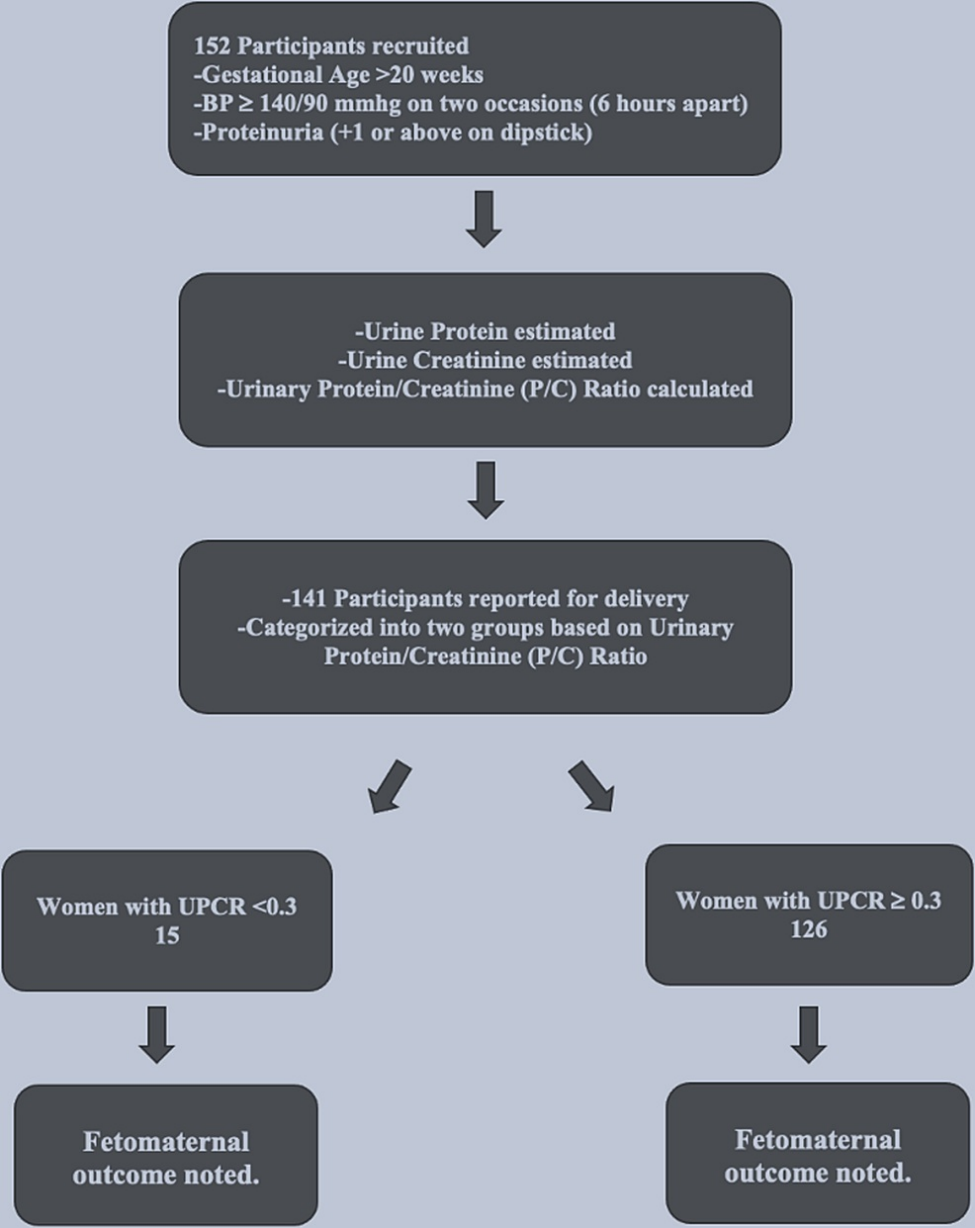
Procedural outflow of the study UPCR: urinary protein-to-creatinine ratio

A total of 152 women participated in the study, and they were followed up till delivery. Out of 152 participants, 141 reported for delivery (11 participants were lost to follow-up) and were observed till discharge (Figure 2).

The total number of women with preeclampsia in the study was 141, of which 15 (10.6%) had UPCR <0.3 while 126 (89.4%) had UPCR ≥0.3. The mean UPCR of preeclamptic women with UPCR <0.3 was 0.21 ± 0.05, while for preeclamptic women with UPCR ≥0.3, it was 0.78 ± 0.38. The overall mean UPCR in the study was 0.72 ± 0.40 (Table 1).

**Table 1:** UPCR in study subjects SD: standard deviation; UPCR: urinary protein-to-creatinine ratio

Study subjects	Mean ± SD	N	%
Women with UPCR <0.3	0.21 ± 0.05	15	10.6
Women with UPCR ≥0.3	0.78 ± 0.38	126	89.4
Total	0.72 ± 0.40	141	100.0

The mean urine creatinine observed in the study was 55.38 ± 23.7 (range: 13.2-229.0). The mean protein value observed in the study was 34.67 ± 16.06 (range: 7-84). The mean UPCR observed in the study was 0.72 ± 0.40 (range: 0.1-2.0) (Table 2).

**Table 2:** Mean urinary protein and creatinine in women with preeclampsia SD: standard deviation; UPCR: urinary protein-to-creatinine ratio

Urine investigation	N	Mean ± SD	Minimum	Maximum
Urine creatinine	141	55.38 ± 23.7	13.2	229.0
Urine protein	141	34.67 ± 16.06	7	84
UPCR	141	0.72 ± 0.40	0.11	2.01

**Figure 3:**
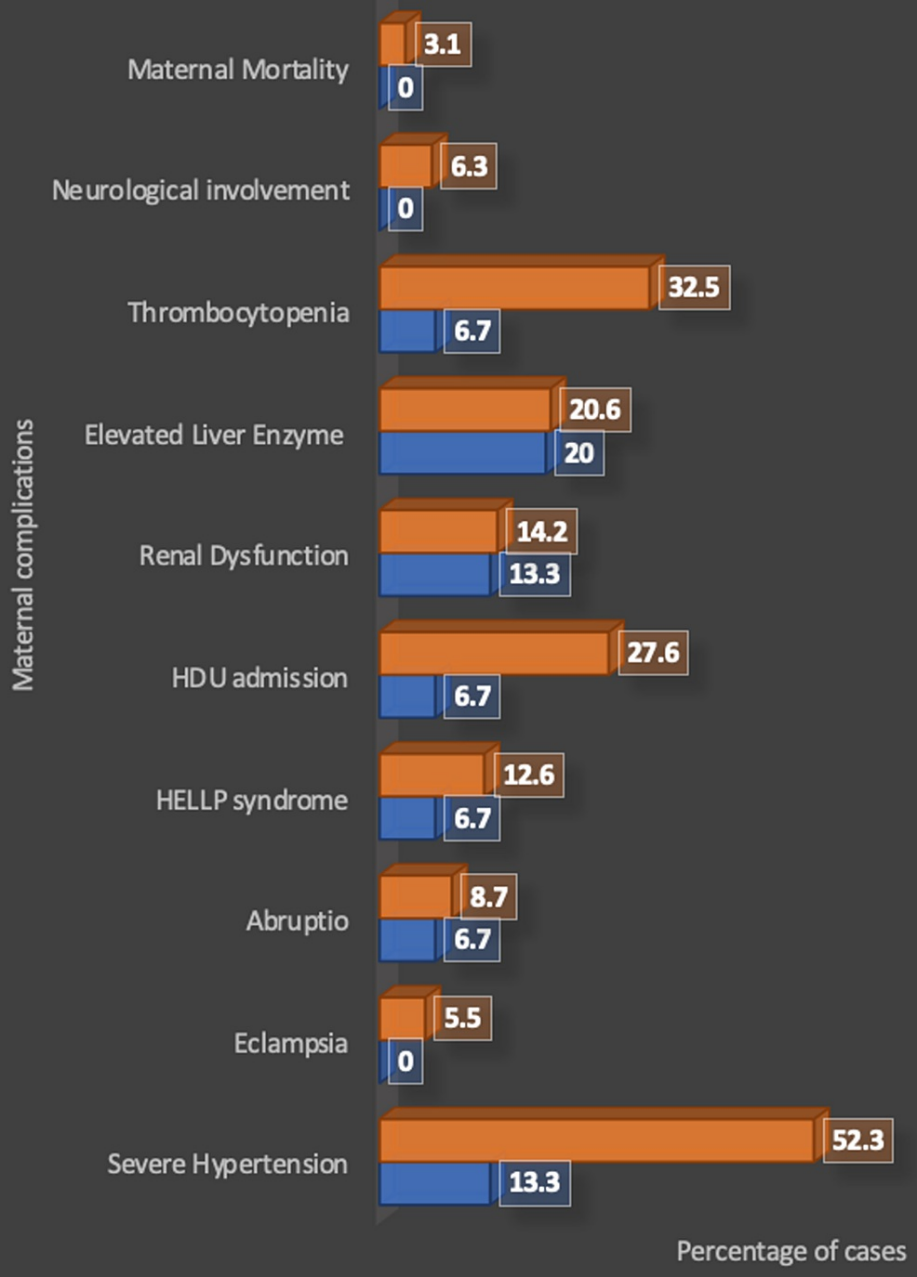
Maternal outcomes and urinary protein-to-creatinine ratio HDU: high-dependency unit; HELLP: Hemolysis, Elevated Liver Enzymes, and Low Platelets

Out of the total 141 women with preeclampsia, development of severe hypertension was observed in 68 (48.2%), eclampsia in seven (5.0%), abruptio placenta in 12 (8.5%), HELLP syndrome in 17 (12.2%); high-dependency unit (HDU) admission was required in 40 (28.4%), gestational diabetes mellitus (GDM) developed in four (2.8%), renal dysfunction in 20 (14.2%), elevated liver enzymes in 29 (20.6%), thrombocytopenia in 42 (29.8%), neurological involvement in eight (5.7%), and maternal mortality was observed in four (2.8%) (Table 3). Eclampsia (seven; 5.5%), GDM (four; 2.8%), neurological involvement (eight; 5.7%), and maternal mortality (four; 2.8%) were only observed in preeclamptic women with UPCR ≥0.3 (Figure 3).

**Table 3:** Maternal outcomes and urinary protein-to-creatinine ratio *Significant p-value HDU: high-dependency unit; HELLP: Hemolysis, Elevated Liver Enzymes, and Low Platelets; UPCR: urinary protein-to-creatinine ratio

Maternal parameters	UPCR <0.3 (n=15)		UPCR ≥0.3 (n=126)		Total (n=141)		χ2	P-value
	N	%	N	%	N	%		
Severe hypertension	2	13.3	66	52.3	68	48.2	8.19	0.01*
Eclampsia	0	0.0	7	5.5	7	5.0	-	-
Abruptio placenta	1	6.7	11	8.7	12	8.5	0.04	0.82
HELLP syndrome	1	6.7	16	12.6	17	12.1	0.06	0.79
HDU admission	1	6.7	39	27.6	40	28.4	3.89	0.04*
Renal dysfunction	2	13.3	18	14.2	20	14.2	0.08	0.77
Elevated liver enzymes	3	20	26	20.6	29	20.6	0.07	0.78
Thrombocytopenia	1	6.7	41	32.5	42	29.8	4.29	0.03*
Neurological involvement	0	0.0	8	6.3	8	5.7	-	-
Maternal mortality	0	0.0	4	3.1	4	2.8	-	-

The mean UPCR for preeclamptic women who developed severe hypertension, eclampsia, renal dysfunction, thrombocytopenia, and required HDU admission showed a statistically significant association (p<0.001). While, the mean UPCR for preeclamptic women who developed abruptio placentae, HELLP syndrome, elevated liver enzymes, neurological involvement, and maternal mortality did not show a significant association (Table 4).

**Table 4:** Maternal outcome and its association with the mean urinary protein-to-creatinine ratio *Significant p-value HDU: high-dependency unit; HELLP: Hemolysis, Elevated Liver Enzymes, and Low Platelets; SD: standard deviation

Maternal parameters		N	%	Mean ± SD	t	P-value
Severe hypertension	Present	68	48.2	0.91 ± 0.43	5.84	<0.001*
	Absent	73	51.8	0.55 ± 0.28		
Eclampsia	Present	7	4.9	1.75 ± 0.18	14.64	<0.001*
	Absent	134	95.1	0.67 ± 0.33		
Abruptio	Present	12	8.5	0.87 ± 0.58	0.93	0.36
	Absent	129	91.5	0.71 ± 0.38		
HELLP syndrome	Present	17	12.1	0.77 ± 0.37	0.51	0.61
	Absent	124	87.9	0.72 ± 0.41		
HDU admission	Present	40	28.3	1.06 ± 0.38	6.86	<0.001*
	Absent	101	71.7	0.59 ± 0.33		
Renal dysfunction	Present	20	14.2	1.18 ± 0.48	4.74	<0.001*
	Absent	121	85.8	0.65 ± 0.33		
Elevated liver enzymes	Present	29	20.6	0.77 ± 0.45	0.65	0.51
	Absent	112	79.4	0.71 ± 0.39		
Thrombocytopenia	Present	42	29.8	1.07 ± 0.42	6.89	<0.001*
	Absent	99	70.2	0.58 ± 0.29		
Neurological involvement	Present	8	5.7	1.04 ± 0.67	1.37	0.20
	Absent	133	94.3	0.71 ± 0.38		
Maternal mortality	Present	4	2.8	0.95 ± 0.55	0.82	0.46

**Figure 4:**
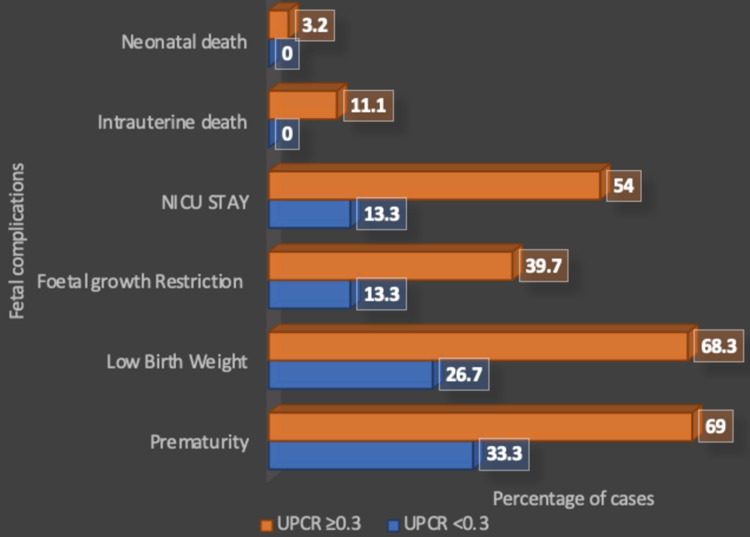
Fetal outcomes and urinary protein-to-creatinine ratio NICU: neonatal intensive care unit; UPCR: urinary protein-to-creatinine ratio

In the present study, out of 141 neonates born to women with preeclampsia, 92 (65.2%) were premature, 90 (63.8%) had LBW, 52 (36.9%) had FGR, 70 (49.6%) were admitted to the NICU, 14 (9.9%) had intrauterine fetal demise, and four (2.8%) experienced neonatal mortality (Table 5). A total of 14 (9.9%) IUDs and four (2.8%) neonatal deaths were observed, and these occurred only in preeclamptic women with UPCR ≥0.3 (Figure 4).

**Table 5:** Fetal outcomes and urinary protein-to-creatinine ratio *Significant p-value NICU: neonatal intensive care unit; UPCR: urinary protein-to-creatinine ratio

Fetal parameters	UPCR <0.3		UPCR ≥0.3		Total		χ2	P-value
	N	%	N	%	N	%		
Prematurity	5	33.3	87	69.0	92	65.2	7.54	0.01*
Low birth weight	4	26.7	86	68.3	90	63.8	10.04	0.01*
Fetal growth restriction	2	13.3	50	39.7	52	36.9	3.99	0.04*
NICU admission	2	13.3	68	54.0	70	49.6	8.85	0.01*
Intrauterine death	0	0.0	14	11.1	14	9.9	-	-
Neonatal death	0	0.0	4	3.2	4	2.8	-	-

The mean UPCR for neonates who were premature, who had LBW, FGR, and required NICU admission showed a strong significant association (p<0.001). The mean UPCR for intrauterine fetal death and neonatal death did not show any statistical significance (Table 6).

**Table 6:** Fetal outcomes and its association with the mean urinary protein-to-creatinine ratio *Significant p-value NICU: neonatal intensive care unit; SD: standard deviation

Fetal parameters		N	%	Mean ± SD	t	P-value
Prematurity	Present	92	65.2	0.87 ± 0.41	8.62	<0.001*
	Absent	49	34.8	0.44 ± 0.18		
Low birth weight	Present	90	63.8	0.87 ± 0.43	7.71	<0.001*
	Absent	51	36.2	0.47 ± 0.18		
Fetal growth retardation	Present	52	36.9	0.95 ± 0.45	5.14	<0.001*
	Absent	89	63.1	0.59 ± 0.30		
NICU admission	Present	70	49.6	0.96 ± 0.40	8.53	<0.001*
	Absent	71	50.4	0.49 ± 0.23		
Intrauterine death	Present	14	9.9	0.67 ± 0.39	0.54	0.59
	Absent	127	90.1	0.73 ± 0.40		
Neonatal death	Present	4	2.8	0.88 ± 0.28	1.11	0.33
	Absent	137	97.2	0.72 ± 0.40		

The sensitivity of UPCR for predicting adverse maternal outcome was 79.37% (95% CI: 71.25-86.06), specificity was 46.67% (95% CI: 21.27-73.41), PPV was 92.59% (95% CI: 88.53-95.29), NPV was 21.21% (95% CI: 12.43-33.81), and accuracy was 75.79% (95% CI: 67.97-82.69). The sensitivity of UPCR for predicting adverse fetal outcome was 76.98% (95% CI: 68.65-84.01), specificity was 13.33% (95% CI: 1.66-40.46), PPV was 88.18% (95% CI: 85.69-90.29), NPV was 6.45% (95% CI: 1.79-20.67), and accuracy was 70.21% (95% CI: 61.94-77.62) (Table 7).

**Table 7:** Diagnostic characteristics of urinary protein-to-creatinine ratio

Urinary protein-to-creatinine ratio	Adverse maternal outcomes		Adverse fetal outcomes	
	%	95% CI	%	95% CI
Sensitivity	79.37	71.25–86.06	76.98	68.65–84.01
Specificity	46.67	21.27–73.41	13.33	1.66–40.46
Positive predictive value	92.59	88.53–95.29	88.18	85.69–90.29
Negative predictive value	21.21	12.43–33.81	6.45	1.79–20.67
Diagnostic accuracy	75.89	67.97–82.69	70.21	61.94–77.62

**Figure 5:**
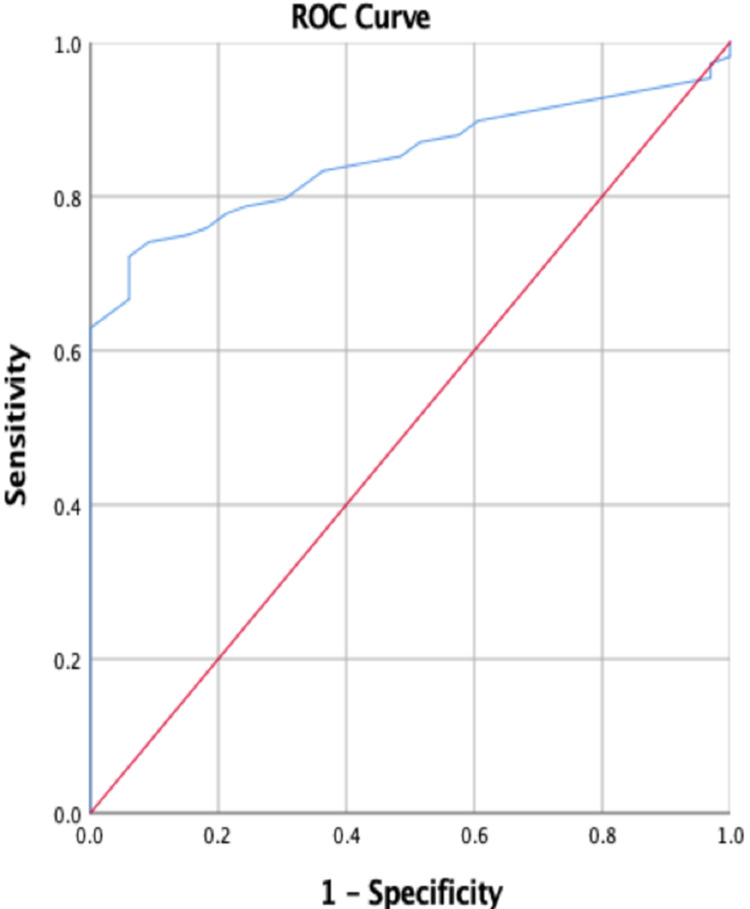
Receiver operating characteristic curve for maternal outcomes

**Figure 6:**
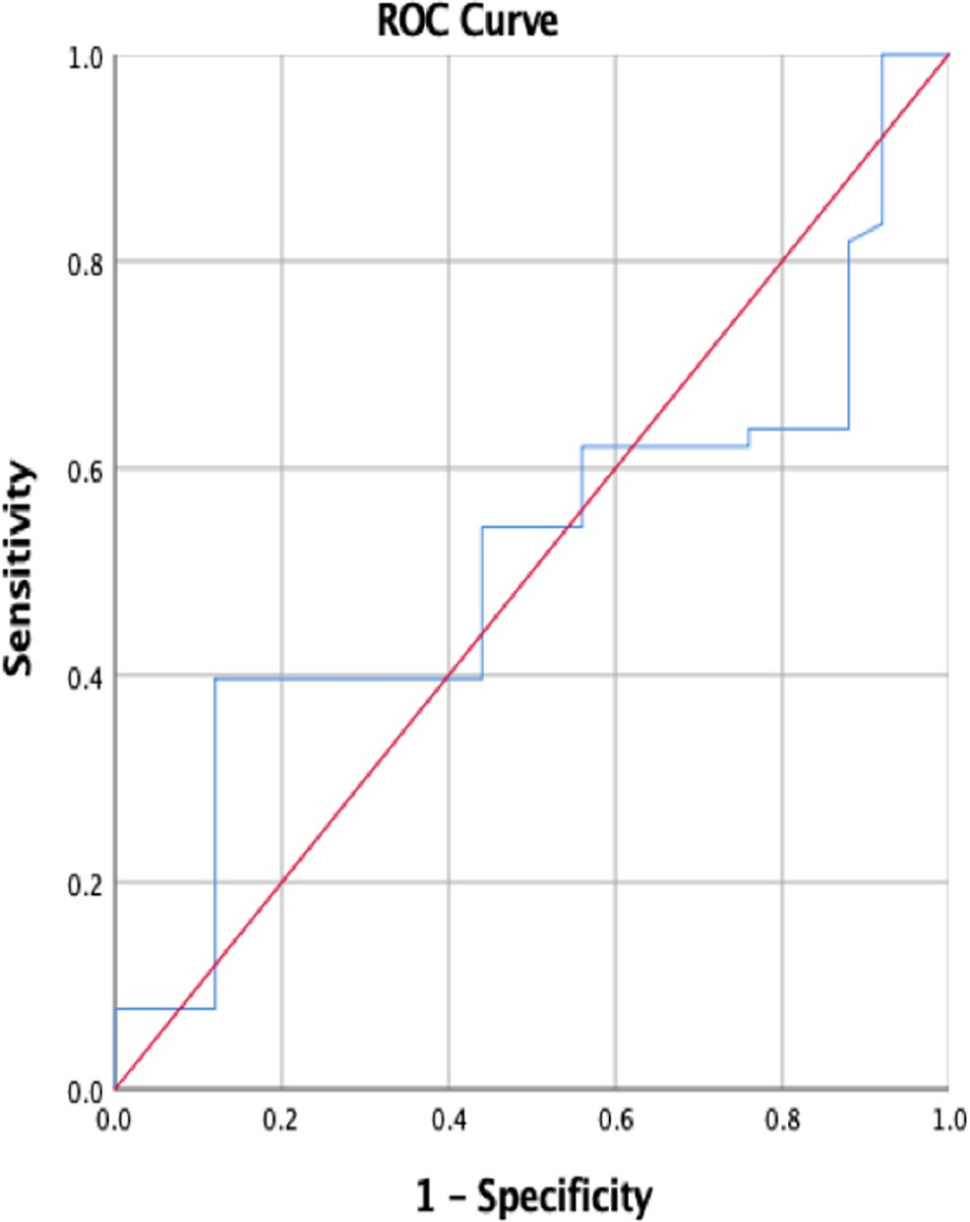
Receiver operating characteristic curve for fetal outcomes

Receiver operating characteristic (ROC) curves above the diagonal line are considered to have a reasonable discriminating ability to predict adverse outcomes in study subjects. UPCR had significant discriminatory power to predict adverse maternal outcomes [area under the curve (AUC): 0.848; 95% CI: 0.878-0.910] (Figure 5) as compared to adverse fetal outcomes (AUC: 0.515; 95% CI: 0.403-0.627) (Figure 6). Among both the parameters, UPCR was a better predictor of adverse maternal outcomes.

## Discussion

Preeclampsia and urinary protein-to-creatinine ratio

Preeclampsia is a multiorgan syndrome defined by two imprecise end-organ involvement markers: hypertension and proteinuria. However, the role of proteinuria in the diagnosis and evaluation of preeclampsia is still a matter of controversy [11]. Proteinuria in preeclampsia is caused by glomerular alterations like endothelial cell enlargement and fenestrae disruption, resulting in increased protein permeability, which includes high-molecular-weight proteins like albumin. A random UPCR of at least 0.3 has been identified as a surrogate marker for proteinuria in preeclampsia.

UPCR is expected to enhance the first screening of proteinuria as Côté et al. (2008) [26] have reported UPCR to be an acceptable test for identifying proteinuria of 0.3 g/day in their systematic review comprising 13 studies. On a similar note, Sanchez-Ramos et al. (2013) [27] have stated that UPCR from urine samples gives good evidence to rule out the presence of statistically significant proteinuria (>300 mg/day) among individuals at risk for preeclampsia as seen in 24 trials with 3,186 aggregate participants. Kumari et al. (2014) [28] studied 200 preeclamptic women and found that the value of spot UPCR was highly linked with 24-hour urine protein for diagnosing significant proteinuria (300 mg/day), the highest discriminatory spot UPCR value being 0.3. Studies conducted by Yamasmit et al. (2004) [29], Saudan et al. (1997) [30], and Dwyer et al. (2008) [12] have shown UPCR to be an effective predictor of significant proteinuria in preeclampsia, whereas studies by Al et al. (2009) [31] and Wheeler et al. (2007) [32] did not find it to be an effective screening tool.

In the present study, out of 141 cases of preeclampsia, 15 (10.6%) had a UPCR <0.3 while 126 (89.4%) had UPCR ≥0.3. This finding was in concordance with various studies conducted to determine the relationship between UPCR and fetomaternal outcomes. Nischintha et al. (2014) [33] included 75 preeclamptic pregnant women in their study and observed that UPCR was <0.3 in nine (12.%) women while it was ≥0.3 in 66 (87.7%) cases. Mahesh and Borgohain (2020) [34] in their prospective study with 70 women to observe fetomaternal outcomes in preeclampsia measured spot UPCR and the results were as follows: 59 (84.2%) had proteinuria ≥300 mg/day while 11 (15.7%) had <300 mg/day, which correlated with the UPCR values. Nigam et al. (2018) [35] in their study of 200 women with preeclampsia found UPCR ≥0.3 in 159 (79.5%) and UPCR <0.3 in 41 (20.5%). The present study observed a mean UPCR of 0.72 ± 0.40 in all 141 preeclamptic women, among which the mean for preeclamptic women with UPCR <0.3 was 0.21 ± 0.05 while the mean for those with UPCR ≥0.3 was 0.78 ± 0.38.

In the study by Bhadarka et al. (2018), a strong correlation was found between UPCR and 24-hour urine protein, suggesting that UPCR appeared to be a viable alternative to 24-hour urine protein excretion, particularly in emergent circumstances. Stefańska et al. (2020) [37] in their study of 100 preeclamptic women suggested that when compared to other regularly used tests, UPCR determination is a reliable, relatively faster, and equally accurate approach for determining proteinuria, and it correlates well with 24-hour urine protein estimations. Hence, spot UPCR is a relatively reliable, accurate, and faster way of quantifying proteinuria. Because of its accuracy, reproducibility, and lack of necessity for a timed 24-hour urine collection, this test has become the commonly recommended approach for assessing proteinuria.

The urinary protein-to-creatinine ratio in preeclampsia and determinants of maternal outcomes

In a retrospective study by Yücesoy et al. (2005) [38] involving 255 cases, 138 patients (54.11%) were reported to have severe preeclampsia with proteinuria >5 gm/day, out of which 28 (10%) experienced eclamptic convulsions, 28 (11%) were demonstrated to have HELLP syndrome, while another 19 (7.5%) had placental abruptions. Kumari et al. (2014) [28] conducted a prospective observational study in patients with preeclampsia to investigate the association between the amount of proteinuria measured by spot UPCR and fetomaternal outcomes. High UPCR (>0.3) was significantly associated with severe hypertension in 44% of cases, eclampsia in 31%, liver enzymes in 21% of cases, and ICU admission in 38% of cases. While no significant association was observed between UPCR and renal insufficiency, thrombocytopenia, neurological involvement (p=0.26), and maternal mortality (p=0.45) were observed in 14%, 23%, 53%, and 3% of cases respectively. The results were in concordance with the present study as a statistically significant association was observed between UPCR ≥0.3 and severe hypertension (52.3%, p=0.01), thrombocytopenia (32.5%, p=0.03), while no such association was observed between UPCR and eclampsia, renal dysfunction (14.2%), and elevated liver enzymes (20.6%). Also, the mean value of proteinuria was statistically significant among patients with renal dysfunction (p<0.001) and thrombocytopenia (p<0.001). However, elevated liver enzymes (p=0.65) showed no significant relationship with proteinuria.

Kim et al. (2017) [39] in their retrospective study found no significant association between levels of proteinuria and eclampsia (p=0.31), renal insufficiency (p=0.33), elevated liver enzymes (p=0.70), and HELLP (p=0.39) but found thrombocytopenia to be significantly related (p=0.01). The incidence of eclampsia was 5.1% in women with significant proteinuria, which is quite similar to the results of the present study as severe hypertension was observed in a significant proportion (48.2%) of women while eclampsia was observed in 5% of the population. The results align with the present study as no such association was observed between UPCR and abruptio placenta (8.5%) and HELLP (12.1%). In the present study, the mean value of proteinuria was also not significant for abruptio placenta (p=0.36) and HELLP syndrome (p=0.61). Lei et al. (2021) [11] in their study observed no cases of eclampsia or maternal mortality in patients with proteinuria >300 mg/day, which was not significant. However, a rate of 3.1% for renal disease and 4.5% for placental abruption was noted among them. In the present study, the mean value of proteinuria was statistically significant among the patients having eclampsia (p<0.001) and severe hypertension (p<0.001). Similar results were seen in the study by Kumari et al. (2014) [28] where the mean value of UPCR was 3.48, 3.92, 2.61, and 2.98 among the patients with severe hypertension (p=0.001), eclampsia (p=0.002), raised liver enzymes (p=0.02), and need for ICU support (p=0.02) respectively, and these findings were highly significant.

Cheung et al. (2016) [40] noted findings where a difference was observed in mean UPCR among 95 cases of preeclampsia who developed severe hypertension (47%, p=0.35), raised liver enzymes (6%, p=0.23), renal insufficiency (6%, p=0.85) and thrombocytopenia (2%, p=0.08), and these results were not significant. Hence, they suggested that proteinuria should not be used to define clinical outcomes in women. Özkara et al. (2018) [41] in their study of 132 preeclamptic women observed that eclampsia was reported in 11.4% of the women with proteinuria more than 3000 mg while no cases were reported among women with proteinuria in the range of 300-1000 mg (p=0.018). The study depicted a significant association between proteinuria and eclampsia as maternal complications among women diagnosed with preeclampsia. Bramham et al. (2013) [42], in a nested case-controlled study involving 946 patients, found that women with preeclampsia and proteinuria between 300-499 mg/24 hours and >500 gm/day had higher rates of eclampsia at 37.7% and 42.9% respectively than women with gestational hypertension and chronic hypertension without proteinuria (p<0.001). Also, higher rates of HELLP, elevated liver enzymes, renal insufficiency, and lower platelets were seen in women with preeclampsia and proteinuria >300 mg/24 hours. Six maternal deaths (2.7%) were noted in women with proteinuria >300 mg/day, which was not a significant association. Bharathi et al. (2019) [43] in their study of 209 antenatal women observed that out of the 157 preeclamptic women, complication rate among women with proteinuria >300 mg/day, >1000 mg/day, and >2000 mg/day were as follows: abruptio placenta: 33.3% vs. 66.7% vs. 0 (p<0.001); HELLP syndrome: 0 vs. 33.3% vs. 66.7% (p<0.001); and eclampsia: 11.7% vs. 77.8% vs. 11.7% respectively (p<0.001), which indicated a very strong significance. Speranza et al. (2019) [44] in their retrospective study stated that renal complications were significantly more common among cases of preeclampsia with severe proteinuria. Martins-Costa et al. (2011) [45] stated that hypertensive pregnant women with a PCR ≥0.3 mg/mg had worse maternal and perinatal outcomes than those with PCR <0.3 mg/mg. Chan et al. (2005) [46] concluded in their study that an adverse maternal outcome was significantly associated with greater spot UPCR at diagnosis with an odds ratio (OR) of 1.003 per mg/mmol. The results were in concordance with the present study as a statistically significant association was observed between UPCR ≥0.3 and HDU admission (27.6%, p=0.04) while no such association was observed between UPCR and eclampsia (5%), neurological involvement (5.7%), or maternal mortality (2.8%). In the present study, the mean value of proteinuria was statistically significant among the patients requiring HDU admission (p<0.001). However, neurological involvement (p=0.20) and maternal mortality (p=0.46) showed no significant relationship with the amount of proteinuria. Nyota et al. (2021) [47] observed that PCR values were disturbed among 38% of the preeclamptic pregnant women who suffered maternal complications; however, it was normal for 5% of the women who suffered complications. This difference was significant, indicating that UPCR levels were associated with maternal complications. However, a systematic review by Thangaratinam et al. (2009) [48], involving 16 primary articles with 6749 women, did not find proteinuria to be an effective predictor of fetomaternal outcomes in preeclampsia. Therefore, in preeclamptic women, increased proteinuria corroborated by raised UPCR values was linked to worsening of maternal outcomes like severe hypertension, eclampsia, thrombocytopenia, renal dysfunction, and requirement for HDU management. Hence, preeclamptic women with high UPCR must be constantly observed and assessed for signs and symptoms, especially of the above-mentioned complications.

The urinary protein-to-creatinine ratio and determinants of fetal outcomes

Despite morbidity mediated by preterm birth, FGR, and LBW, preeclampsia is independently linked to adverse neonatal outcomes. Even if all infants could be delivered at term, owing to preeclampsia, there remains an increased neonatal risk. Because delivery is the only option to stop preeclampsia from progressing, this risk may be linked to disease severity.

In a retrospective cohort study, Chan et al. (2005) [46] suggested that an increased risk of adverse fetal outcome was associated with a higher spot UPCR [OR: 1.44 per log (mg/mmol)]. A study by Kumari et al. (2014) [28] also indicated a higher mean UPCR of 2.69 ± 0.86 in preterm live births (44%) in comparison to 2.14 ± 1.05 in full-term live births (43%), suggesting a significant association between UPCR and prematurity (p=0.001). Also, 22% of the small-for-gestational-age newborns (FGR) had a mean UPCR of 3.08 while those with no FGR had a mean UPCR of 2.98, showing no significant association between FGR and UPCR (p=0.81). A significant association was observed between UPCR and birth weight (mean UPCR: 3.42, p=0.04), low APGAR (activity, pulse, grimace, appearance, and respiration) score at five minutes (mean UPCR: 3.98, p=0.001), and NICU admissions (UPCR of 3.21, p=0.03). Of note, a 23% perinatal mortality rate was seen in their study, which included 13% stillbirths (p=0.27) and 10% early neonatal deaths (p=0.30). The mean UPCR in those with perinatal mortality was 3.80 as compared to 2.76 in those with no mortality, which was found to be significant (p=0.001). The present study found a neonatal mortality rate of 3.2% (n=4) in women with UPCR ≥0.3 while no mortality was observed in the group with UPCR <0.3. When the mean UPCR for both the groups was compared (with and without neonatal mortality), no significant association was found (p=0.33). The present study also observed 11.1% IUDs in women with UPCR ≥0.3 while no deaths were reported in groups with UPCR <0.3. When the mean UPCR for both the group with IUDs and that without IUDs was compared, no significant difference was observed (p=0.59).

Bramham et al. (2013) [42] also noted higher rates of adverse fetal outcomes in preeclamptic women with proteinuria >300 mg/day and >500 mg/day, with preterm births at 25% and 61.5%, small-for-gestational-age infants at 40% and 52.5%, and perinatal deaths at 3.3% and 2.5% respectively, as compared to chronic and gestational hypertensive women without proteinuria. Another retrospective cohort study conducted by Guida et al. (2018) [49] among 293 patients reported that 22.5% of the neonates born were preterm in the group of women with massive proteinuria, indicating that massive proteinuria in preeclamptic women leads to increased risk of preterm birth. Bharathi et al. (2019) [43] found that among 157 preeclampsia subjects, 85 patients had adverse fetal outcomes, with 20% prematurity (p=0.006) in subjects with proteinuria >300 mg/day, concluding that fetal adversities increase with higher proteinuria. The present study had similar findings since the majority of the pregnant women (55.3%) delivered preterm live babies. When a comparison between the two groups was made, the majority of the newborns were preterm (57.9%) in the group with UPCR ≥0.3 while the majority were full-term (66.7%) in the group with UPCR <0.3, revealing a significant association between preterm births and UPCR (p=0.02). In another retrospective study, Lei et al. (2021) [11] found higher rates of prematurity at 10.9% vs. 34% (p=0.009) and stillbirths at 4.7% vs. 17% (p=0.002), while FGR at 7.8% vs. 11.3% (p=0.563) and LBW at 6.2% vs. 7.4% (p=0.59) were seen in preeclamptic women with proteinuria >300 mg/day and >2000 mg/day respectively, as compared to those with proteinuria <300 mg/day. Similarly, in the present study, the rate of prematurity was 69% vs. 33 (p=0.01) in the group with UPCR ≥0.3 and that with UPCR <0.3 respectively, indicating a significant association between preterm births and UPCR ≥0.3. Xiong et al. (1999) [50] in their study on the impact of preeclampsia on fetal growth found that after adjustment for duration of gestation and other confounders, preeclampsia and severe hypertension increased the risk of FGR. Hence, the present study suggests timely counseling for preeclamptic women with high UPCR about premature newborns and the need for a team of specialists along with NICU support for the management of such infants.

Shinar et al. (2016) [51], in a retrospective cohort study, also concluded that greater levels of proteinuria were associated with increased risk for FGR (p=0.03) and lower APGAR score at five minutes (p=0.04). In the present study, the rate of FGR was 39.7% vs. 13.3% (p=0.04) in the group with UPCR ≥0.3 and that with UPCR <0.3 respectively, indicating a significant association between higher UPCR and FGR. The present study also observed a significant association between lower APGAR at five minutes and higher UPCR, as a score of 0-6 was observed in a higher percentage of newborns (58.7%) belonging to the group with UPCR ≥0.3 in comparison to the group with UPCR <0.3 (13.3%); the majority, i.e., 86.7%, had APGAR score >7 in the group with UPCR <0.3. In the study by Bharathi et al. (2019) [43], 85 patients with preeclampsia who had adverse fetal outcomes showed a 60% FGR rate (p=0.006), 80.9% showed LBW (p<0.001), and a perinatal death rate of 17.5% was observed (p=0.006) when proteinuria was >300 mg/day, indicating that greater proteinuria increases the risk of fetal adversity. In a study by Jan et al. (2017) [52], where the mean birth weight of newborns was 2479 ± 469.09 gm in patients with preeclampsia, the mean difference of birth weight between those with gestational hypertension (without proteinuria) and preeclampsia was found to be statistically significant. Similar results were seen in the present study where 49.6% of the total study population delivered LBW neonates. The present study found a significant association between UPCR and LBW as mothers of 68.3% [3.2% were extremely low birth weight (ELBW), 11.1% were very low birth weight (VLBW), and the reaming 54% were LBW] of the LBW newborns had UPCR ≥0.3 while 73.3% of those with normal birth weight newborns had UPCR <0.3 (p=0.01).

Özkara et al. (2018) [41] have reported that the mean birth weight of neonates showed a decreasing trend as proteinuria increased above 300 mg; the birth weight of 2521 ± 640 gm in neonates born to women with proteinuria in the range of 300-1000 mg/day was significantly higher than 2230 ± 732 in those born to women with proteinuria >3000 mg/day. The study depicted a significant association between LBW and high levels of proteinuria (p=0.054). NICU admission was significantly higher for women who had proteinuria above 3000 mg in comparison to women having proteinuria in the range of 300-1000 mg, indicating a significant relationship (p=0.015). According to Cheung et al. (2016) [40], spot UPCR was significantly greater among neonates who were admitted to the NICU (p=0.002). All the results from other studies are in concordance with the present study as NICU stay was higher for neonates born to mothers with UPCR ≥0.3. However, Nischintha et al. (2014) [33] observed dissimilar results as no significant association was observed between UPCR and birth weight (p=0.645), but this could be attributed to the small sample size of the study, which included only 75 participants. The present medical evidence still remains divided, with some studies suggesting that in preeclampsia, neonatal adversities are an outcome of prematurity rather than the disease itself. Hence, more in-depth studies are needed to establish a relationship between the two. The present study recommends timely referrals of such patients to an established high-level NICU for appropriate management.

In the study by Kim et al. (2017) [39], 9.8% of the neonates with APGAR score <7 at five minutes were born to mothers having severe proteinuria in comparison to 7.7% of the newborns of women with mild proteinuria (<300 mg/day), which was not significant (p=0.55). They also reported a significantly higher NICU admission with cases of severe proteinuria (>5 g/day) (52.2%) in comparison to mild proteinuria (2 g/day) (25.3%) (p=0.001). Hence, the present study suggests that preeclamptic women with high UPCR are more likely to have FGR and may give birth to small-for-gestational-age neonates, who might have lower APGAR scores and a greater requirement for NICU admissions, hence requiring a maternal-fetal medicine specialist or neonatologist and on-hand resuscitation measures during delivery. These patients must be counseled effectively for all plausible outcomes. These patients might also require guidance and support to deal with such adversities.

Predictive value of the urinary protein-to-creatinine ratio for fetomaternal complications

The relationship between proteinuria and poor fetomaternal outcomes has been explored by several researchers. Elevated protein excretion in preeclamptic women is often related to poor maternal as well as fetal outcomes. Urine protein levels are a simple yet effective way of identifying women with preeclampsia who are prone to developing unfavorable fetomaternal outcomes, which has been harnessed by this tool: UPCR. Various studies have reported on the efficacy of UPCR as a predictor of fetomaternal complications in preeclampsia (Table 8). The present study was conducted on 141 pregnant women with preeclampsia and, using a cut-off value of 0.3, UPCR was found to have a sensitivity of 79.35% and 76.98%, specificity of 46.67% and 13.33%, PPV of 92.59% and 88.18, and NPV of 21.21% and 6.45% for maternal and fetal complications respectively.

**Table 8:** Diagnostic accuracy of urinary protein-to-creatinine ratio as reported by various studies

Author	Year	Location	Sample	Sensitivity	Specificity
Côté et al. [26]	2008	-	13 studies	83.6%	76.3%
Cheung et al. [40]	2016	China	120	82%	79%
Bhadraka et al. [36]	2018	India	100	92%	85%
Nigam et al. [35]	2018	India	200	83.6%	100%
Stefańska et al. [37]	2020	Poland	88	100%	89%

Even though the sensitivity and specificity were not that high in our study, PPV was promising, and this prompts us to recommend the use of UPCR as a predictive tool for preeclampsia in a rural population with a relatively high prevalence of preeclampsia. Identifying these women would also enable better counseling, vigilant follow-up, raising of awareness among women at high risk, and initiation of appropriate therapies at an early stage.

This study has some limitations. Firstly, this was a hospital-based study with limited sample size, and hence the results cannot be generalized to wider community settings. Further well-planned, community-based, and multicentric studies must be conducted to better assess the value of UPCR as a predictor of abnormal fetomaternal outcomes, so as to include its use in the care bundle for high-risk pregnancies complicated by preeclampsia.

## Conclusions

Based on our findings, a high UPCR is seen in approximately four out of five women with preeclampsia, especially in those who are pregnant for the first time and have a high BMI. Women with preeclampsia having high UPCR are at a higher risk of developing maternal complications like severe hypertension, eclampsia, renal dysfunction, and thrombocytopenia, indicating the need for frequent and vigilant antenatal surveillance, and they are more likely to need monitoring and management in obstetric HDUs. Thus, these women require higher vigilance in the peripartum period with counseling for emergency operative delivery. Babies born to women with preeclampsia having high UPCR have a higher likelihood of FGR, premature birth, LBW, poorer APGAR scores, and need care in NICUs, highlighting the importance of institutional delivery at tertiary care centers equipped with dedicated maternal and newborn care units. To summarize, UPCR is a simple laboratory tool to predict abnormal fetomaternal outcomes in preeclampsia with good sensitivity and PPV and can be used as an adjunct to assist in clinical decisions.
